# Can We Treat Neuroinflammation in Alzheimer’s Disease?

**DOI:** 10.3390/ijms21228751

**Published:** 2020-11-19

**Authors:** Sandra Sánchez-Sarasúa, Iván Fernández-Pérez, Verónica Espinosa-Fernández, Ana María Sánchez-Pérez, Juan Carlos Ledesma

**Affiliations:** Neurobiotechnology Group, Department of Medicine, Health Science Faculty, Universitat Jaume I, 12071 Castellón, Spain; sarasuad@uji.es (S.S.-S.); ivfernan@uji.es (I.F.-P.); veronica.espinosa@uji.es (V.E.-F.)

**Keywords:** Alzheimer’s disease, neuroinflammation, insulin resistance, nutraceuticals, endocannabinoid system, gut microbiota

## Abstract

Alzheimer’s disease (AD), considered the most common type of dementia, is characterized by a progressive loss of memory, visuospatial, language and complex cognitive abilities. In addition, patients often show comorbid depression and aggressiveness. Aging is the major factor contributing to AD; however, the initial cause that triggers the disease is yet unknown. Scientific evidence demonstrates that AD, especially the late onset of AD, is not the result of a single event, but rather it appears because of a combination of risk elements with the lack of protective ones. A major risk factor underlying the disease is neuroinflammation, which can be activated by different situations, including chronic pathogenic infections, prolonged stress and metabolic syndrome. Consequently, many therapeutic strategies against AD have been designed to reduce neuro-inflammation, with very promising results improving cognitive function in preclinical models of the disease. The literature is massive; thus, in this review we will revise the translational evidence of these early strategies focusing in anti-diabetic and anti-inflammatory molecules and discuss their therapeutic application in humans. Furthermore, we review the preclinical and clinical data of nutraceutical application against AD symptoms. Finally, we introduce new players underlying neuroinflammation in AD: the activity of the endocannabinoid system and the intestinal microbiota as neuroprotectors. This review highlights the importance of a broad multimodal approach to treat successfully the neuroinflammation underlying AD.

## 1. Introduction

Alzheimer’s disease (AD) is the most common type of dementia, and is characterized by a progressive loss of memory, visuospatial and complex cognitive abilities, such as language and reasoning, which ultimately lead to a total inability to perform any type of daily activity [1,2]. Oftentimes, the patient presents comorbid psychopathologies, including depression, psychosis, anxiety, aggressive and antisocial behavior. Histologically, AD has been traditionally characterized by the appearance of neurofibrillary tangles (NFTs) and amyloid plaques [3]. NFTs are the intracellular aggregation of hyperphosphorylated Tau, a microtubule-associated protein that provides axonal cytoskeleton stability. Under pathological conditions (e.g., neuroinflammation and insulin resistance), Tau undergoes hyperphosphorylation, and consequently, conformational changes that reduce its affinity for microtubules [3], leading to neurodegeneration [4]. Misfolded Tau can spread via migration to neighbor healthy neurons, worsening the condition [4,5]. Senile amyloid plaques are formed by amyloid β (Aβ) peptide accumulation [6]. The old amyloid hypothesis to explain AD postulates that soluble Aβ oligomers, and Aβ deposits in plaques, together with NFTs are ultimately responsible for neuronal death.

**Figure 1:**
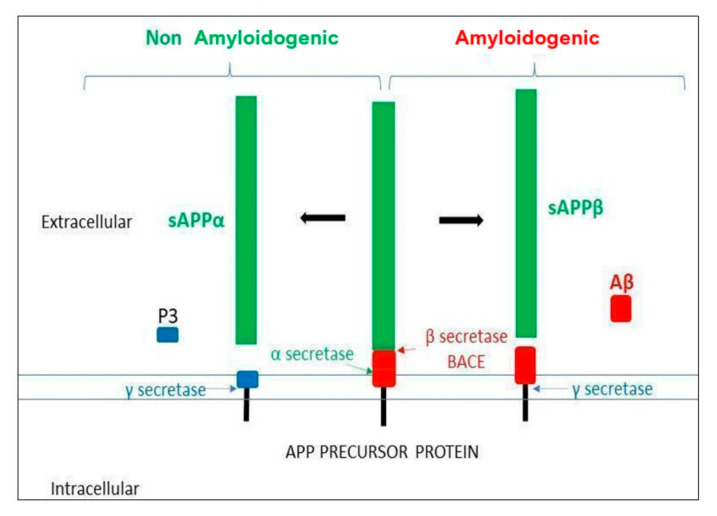
Amyloid precursor protein (APP) processing. The α and γ secretases are involved in the non-amyloidogenic pathway, whereas the β and the γ secretases are involved in the amyloidogenic pathway, generating the Aβ toxic oligomer.

Aβ-peptide is generated from the amyloid protein precursor (APP) proteolysis. APP is a transmembrane glycoprotein expressed in a wide variety of cells and located on chromosome 21 (21q21.3, in mammals) and it undergoes proteolysis by secretases in two possible pathways (see Figure 1). The amyloidogenic pathway starts by the action of the β-site APP-cleaving enzyme 1 secretase (BACE 1), followed by the action of the γ-secretase (presenilin). This sequential cleavage releases different lengths of Aβ peptides (39–42aa), depending on γ-secretase action. Long peptides (Aβ_42_) are more prone to aggregation. The non-amyloidogenic pathway starts by the α-secretase action followed, as above, by γ-secretase, where no pathological peptides are generated. In healthy conditions, both pathways would compete in APP proteolysis [7], and the clearance of amyloid products is carried out by microglia, the resident brain macrophages. An imbalance between the formation and clearance of Aβ peptides results in their aggregation and accumulation in amyloid plaques [8].

Soluble Aβ oligomers can also be neurotoxic, since they induce intracellular oxidative stress and synaptic dysfunction [9,10] through the aberrant interaction with numerous receptors (NMDA, AMPA, acetylcholine, insulin, BDNF and receptors for advanced glycosylation end products; for a review, see [6]). Since deposition of Aβs in amyloid plaques have been observed in other dementias and in non-demented aged people, it is nowadays only considered a specific AD hallmark if it is observed in addition to other signs, such as NFTs, neurodegeneration, insulin resistance and neuroinflammation [11,12]. Importantly, inflammation and insulin resistance start the pathological process years before the appearance of AD’s first clinical symptoms [13,14,15].

## 2. Neuroinflammation in AD

Neuroinflammation is a process regulated by brain resident macrophages, the microglia cells, which are required to recognize and eliminate any toxic component in the central nervous system (CNS) (for a review, see [16]). Microglia has a high capacity for mobility, and they can switch between two different phenotypes, M1 and M2, characterized by a different morphology and cytokine profile. The M2 phenotype is the resting type that actively monitors the brain in healthy conditions [17]. The switch to M1 begins with the recognition of the pathogen-associated molecular patterns (PAMPs) or the damage-associated molecular patterns (DAMPS) by the pattern recognition receptors (PRRs). This includes the ‘toll-like receptors’ (TLRs) in microglia membrane (both plasma and endosomal membrane), cytoplasmic NOD-like receptors (NLR), intracellular retinoic acid-inducible gene-I-like receptors and transmembrane C-type lectin receptors (for a review, see [18]). PAMPS and DAMPS range from bacterial wall components, the lipopolysaccharides (LPS) and virus capsid proteins, to debris released by dying cells and Aβ oligomers [19]. The activation of this system, the so-called inflammasome, initiates the inflammatory cascade, which results in the secretion of several pro-inflammatory cytokines, such as tumor necrosis factor-α (TNF-α), interferon-γ (IFN-γ) and interleukins 1β, 6 and 18 (IL-1β, IL-6 and IL-18, respectively). Pro-inflammatory cytokines purpose is to orchestrate the neutralization and elimination of toxic molecules and/or cellular debris. In normal conditions, once the toxic stimuli have been cleared, microglia swifts to the anti-inflammatory (M1) phenotype and secretes anti-inflammatory cytokines such as interleukins 4, 10 and 18 (IL-4, IL-10 and IL-18, respectively), brain-derived neurotrophic factor (BDNF) or nerve growth factor (NGF), whose role is to terminate the innate immune response and contribute to restore the synaptic function. However, under pathological conditions, microglia cells do not go back to their resting state, thus causing a chronic inflammation process, with the overproduction of pro-inflammatory cytokines and reduction of neuroprotective factors that in sustained situations become highly toxic, leading to neurodegeneration [20].

Therefore, the chronic neuroimmune system activation underlies the initiation and progression in many dementias, and surely, is involved in the late onset of AD [21,22,23]. Not only Aβ activates the microglia [24], but also misfolded Tau interaction with microglia triggers inflammation [25]. The elimination of the microglial receptor, NLR family pyrin domain containing 3 (NLRP3) has shown to reduce brain Aβ levels in rodent models of AD [26,27]; since then, NLRP3 inflammasome has been deeply studied and characterized in AD [28,29]. In addition to the neurological symptoms, neuroinflammation also underlies the psychiatric signs associated with AD, and for that reason, targeting neuroinflammation has also been proposed to treat those comorbid disturbances [30].

According to the neuroinflammation hypothesis underlying AD, there is a lower incidence of AD among users of chronic non-steroidal anti-inflammatory molecules (NSAIDs) [31,32]. NSAIDs inhibit mostly the cyclooxygenase (COX) activity, which synthesizes prostaglandin (PG) from arachidonic acid. At least two isoforms have been described, COX-1 and 2. COX-1 is expressed constitutively; in contrast, COX-2 is induced by inflammation and cellular stress, increasing PG production [33]. Anti-inflammatory compounds, inhibiting COX activity, Naproxen and Celecoxib have been tested in clinical trials against AD. Naproxen, a non-selective COX inhibitor was administered (220 mg/twice day for two years) to 195 pre-symptomatic AD subjects (aged 55+) with a familial history of AD. The progression of the disease was evaluated with the Alzheimer’s Progression Score (APS). Naproxen reduced the rate of the APS, though not significantly [34]. Celecoxib, a selective COX-2 inhibitor, was administrated (200 mg/twice day for 2 years) in 677 pre-symptomatic subjects (70+) with at least one first-degree relative with AD. No improvement in the cognitive symptoms in the Alzheimer’s Disease Anti-inflammatory Prevention Trial (ADAPT) in the AD patients compared to the placebo group was found [35]. None of these clinical trials analyzed inflammation biomarkers; therefore, these studies cannot test the neuro-inflammation hypothesis underlying AD progression. In addition, these clinical data would shift the focus to different inflammation pathways, other than the COX-PG pathway.

Furthermore, the specific TNF-α inhibitor, Etanercept, was evaluated in a small group of 41 AD patients (55+) with mild to severe AD (SMMSE score between 10 and 27), to test its anti-inflammatory effect and subsequent improvement of cognitive function. The weekly 50 mg subcutaneous administration was well tolerated; however, after 24 weeks of treatment, Etanercept did not show significant beneficial effects in cognition, behavior, systemic cytokine levels or global function compared to the placebo-treated group [36]. The failure of this clinical trial involves many factors, including insulin resistance [37]; thus, inhibiting specifically the TNF-α action may not be sufficient to counteract the inflammasome activity, and hence, to effectively prevent disease, perhaps due to the short period of time of assays,. In Table 1, the clinical studies testing anti-inflammatory molecules with potential therapeutic value for Alzheimer disease treatment are presented.

## 3. Targeting Insulin Resistance to Treat AD

The late onset of AD is strongly associated with insulin resistance; in fact, AD has been often recognized as Type 3 diabetes [38]. Several situations can bring about insulin resistance: metabolic syndrome caused by high fat diet, sedentarism, obesity, genetic predisposition and neuroinflammation [39]. Insulin resistance increases Tau aberrant phosphorylation, the expression of APP and the formation of Aβ oligomers and its deposition. In addition, insulin resistance augments oxidative and endoplasmic reticulum stress, mitochondrial dysfunction and pro-inflammatory cascades [40]. Not surprisingly, Type 2 Diabetes mellitus (T2DM) has been associated with cognitive impairment [41].

For this reason, administration of intranasal (IN) insulin has been considered as a potential therapeutic strategy against AD. A systematic review on this strategy concluded that whereas IN insulin administration showed improvement in verbal memory and story recall, it was not effective on other aspects of cognition. Interestingly, the authors conclude that the treatment is affected by the Apoe4 isoform, where Apoe4 (–) patients displayed more benefits compared to Apoe4 (+) patients. This systematic review concluded that current data do not demonstrate that IN insulin can be used as a treatment for dementia of AD or mild cognitive impairment (MCI), although it is very safe, not interfering with systemic glucose levels. Most importantly, these data support that proper stratification by disease stage, Apoe4 carrier status and different types of insulin must be considered for a better therapeutic effect [42].

**Figure 2:**
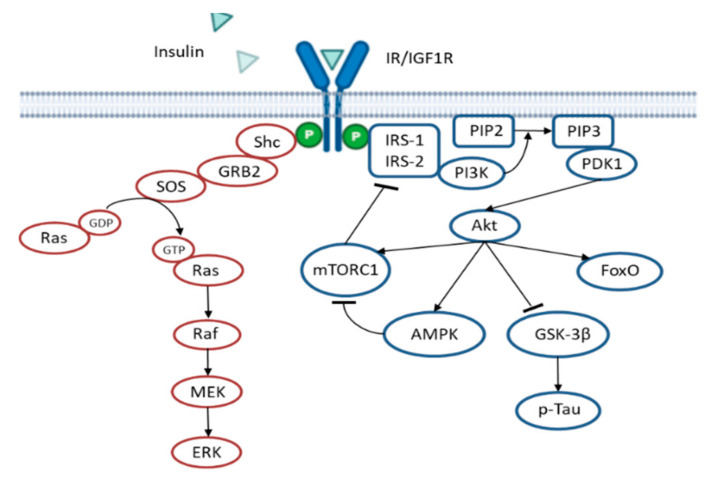
Insulin signaling cascade. The scheme shows the negative feedback mechanism that mTORC1 exerts over IRS1/2. Activation of AMPK inhibits mTORC1, thus improving insulin signaling. In pathological situations, insulin resistance reduces Akt activity, leading to higher GSK-3β activity and subsequent Tau hyperphosphorylation, an important hallmark of AD.

In Insulin resistance situations, the administration of insulin is not effective in the long term; thus, other treatments have been developed instead to enhance the insulin sensitivity, rather than overload the system with insulin. These strategies include the activation of adenosine monophosphate (AMP)-activated protein kinase (AMPK). AMPK activation inhibits the mammalian target of rapamycin (mTOR)/p70 ribosomal S6 kinase (p70S6K) activity [43]. The mTOR/p70S6K pathway is activated by insulin and phosphorylates the insulin receptor substate 1 (IRS1) on serine residues as a negative feedback loop to reduce insulin signaling [44,45] (see Figure 2). Interestingly, AMPK activity displays an anti-inflammatory effect, decreasing inflammatory cells proliferation and their adhesion to the blood vessel endothelium. AMPK activity also reduces amyloidogenesis, Tau hyperphosphorylation and the activation of autophagic degradation [43]. In agreement with this, a pilot study in non-diabetic subjects (aged 55–80 years) diagnosed with MCI, Metformin (an AMPK activator) administration, ameliorated learning, memory and attentional abilities, evaluated by the Paired Associates Learning (PAL) scale and DMS Percent Correct Simultaneous. Despite the improvement in behavior, no changes in the cerebrospinal fluid (CSF) of Aβ_42_, and total or phosphorylated Tau levels were found [46], further suggesting the idea that the amyloid hypothesis does not accurately explain AD.

Milk-derived proteins have also been proposed as possible antioxidant and anti-inflammatory compounds, given their capability to reduce insulin resistance. For example, lactoferrin (a multifunctional iron-binding glycoprotein) administration increases insulin sensitivity in adipose tissue explants from obese subjects [47,48]. Indeed, Lactoferrin antioxidant function is highly dependent on its iron binding capacity [49]. In metabolic syndromes, iron accumulation is considered an important factor underlying insulin resistance and oxidative stress; accordingly, iron-chelators have a positive effect ameliorating the physiopathology of obesity. Lactoferrin therapeutic potential against AD was demonstrated in a pilot study with AD patients. Short-term administration of lactoferrin (250 mg/day for three months) reduced serum oxidative levels and neuroinflammatory markers, and regulated neurotransmitters serum levels concomitant with improved cognitive performance, compared to control [50].

Moreover, deficiency in micronutrients such as vitamin B12 (critical for mental health [51]) has been associated with insulin resistance [51,52,53]. Interestingly, combined treatment of folic acid and vitamin B12 has been shown to improve AD cognitive performance in a randomized trial of 240 patients diagnosed with MCI for 6 months, concomitant with a reduction in serum inflammatory markers [54]. Additionally, Vitamin B12 in combination with anti-psychotic drugs (Risperidone and Quetiapine) reduced blood levels of the pro-inflammatory cytokines IL-8 and TNF-α and augmented the expression of the anti-inflammatory cytokine TGF-β, compared to non-treated AD patients [55]. The same medication formulation was tested in psychotic patients for the expression of the Cluster of Differentiation 68 (CD68), a protein expressed by monocytes and macrophages that has been shown to correlate positively with psychotic symptoms in AD patients. This treatment reduced CD68 expression [56], and therefore, has been proposed as a good strategy against AD. In addition, CD68 has been shown to bind and internalize oxidized Low-Density Lipoprotein (oxLDL), a cholesterol carrier [57], suggesting a relationship of CS68 with intracellular lipid accumulation and atherogenesis. 

In this line of research, pharmacological treatments used to treat other diseases, such as hypertension (i.e., calcium channel blockers) [58,59] or hypercholesterolemia (i.e., statins) [60,61], were postulated as therapeutic agents against AD, given their alleged anti-inflammatory and insulin sensitizing properties. The results from a randomized clinical trial demonstrated that, for instance, the calcium channel blocker nilvadipine has no beneficial effects in a clinical trial against treating AD [62]. On the other hand, statins’ potential therapeutic effect against Alzheimer seems controversial. Simvastatin has been shown to improve memory deficits only at higher doses (80 mg/daily for 18 months) in small groups of patients (50+) [63]; lower doses in larger groups, even though they were efficient in lowering lipid levels, did not ameliorate memory performance [64]. Another statin has been evaluated, artovastatin. In an 18 months clinical trial in dyslipidemic patients, although artovastatin effectively corrected dyslipidemia and inflammatory markers, cognitive function was not evaluated in the study [65]; furthermore, a randomized clinical trial demonstrated no beneficial effects of artovastatin treatment on AD patients’ symptoms [66]. These data conclude that although there is a promising therapeutic evidence in correcting dyslipidemia with statins treatment in AD, more studies are needed to establish their therapeutic applications in AD patients.

Taken together, all the clinical data presented in this section (Table 2) suggest that targeting insulin resistance is a promising strategy to fight AD; however, longer longitudinal studies and larger cohort studies with stratified patients will provide a better profile of successful treatment.

## 4. Nutraceuticals as a Treatment of AD

In the last years, several molecules isolated from plants, also known as nutraceuticals, have been proposed as useful tools for ameliorating cognitive functions and reducing neuroinflammation in animal models of AD.

Because of its anti-oxidant and anti-inflammatory properties, polyphenol compounds belong to the most investigated nutraceuticals to treat human pathologies. Among them, curcumin (the active component of *Curcuma longa*, or turmeric) is traditionally prescribed in Ayruvedic medicine [68], given its remarkably anti-inflammatory effect [69,70,71]. Despite the preclinical studies providing promising results, there is still no clear evidence of its application to AD patients. One study showed improvement in cognitive function when administered to older population compared to control group [72]. However, when it was tested in a clinical trial with AD patients, the study could not evaluate its efficacy, since the control group did not show a decline in cognitive function during the time of the study [73]. Thus, further testing needs to be carried out before concluding the beneficial effect of curcumin on human AD patients.

Resveratrol (RV), a polyphenol no flavonoid found in fruits, including nuts, berries and grape skin, is a Sirtuin activator, stimulates cell survival and prevents apoptosis, neuroinflammation and oxidative stress [74,75]. In clinical studies, RV administration (up to 1 g/twice day orally for 52 weeks to 119 patients diagnosed with mild to moderate AD), was found to reduce CSF and plasma inflammatory markers and Aβ_42_, together with a reduced decline in mini-mental status examination (MMSE), and ADCS-ADL scores [76,77]. Hence, RV based therapy holds a very promising strategy to treat AD symptoms.

Other polyphenols have also been shown to obtain promising results in preclinical models of AD. Among them: Epigallocatechin gallate (EGCG) (green tea) is an α-secretase activity inhibitor [78,79]; Ferulic acid, found in grains, fruits, and vegetables, has been tested alone [80] or in combination with EGCC [81]; Silibinin (herb milk thistle; *Silybum marianum*) [82]; Apigenin, found in vegetables, fruits, herbs and plant-based beverages [83,84]; Puerarin, a Chinese herbal medicine [85,86]. Nevertheless, to our knowledge, in spite of the therapeutic potential of these compounds, they have not been tested in human patients.

Polyunsaturated fatty acids (PUFAs) (contained in blue fish and vegetables such as corn, soybeans, sunflowers, pumpkins, walnuts and others) have been postulated to have a protective role against dementia [87,88]. In contrast to PUFAs, Palmitoylethanolamide (PEA), a peroxisome proliferator-activated receptor alpha (PPAR-α) agonist, exacerbates oxidative stress and amyloid burden in AD mouse models [89,90]. However, despite the promising results in preclinical models, the multidomain clinical trial MAPT study showed that PUFA supplementation had no significant effects on cognitive decline over three years in elderly people with memory complaints [91]. Another nutraceutical commonly used as a herbal medicine and food supplement given its anti-inflammatory properties is ginsenoside (ginseng saponin) [92]. Ginsenosides are amphipathic compounds, acting as partial agonists of steroid hormone receptors, regulating metabolism and inflammation [93]. In a pilot study with 40 patients diagnosed with moderate AD, Ginseng administration (1.5–4 g/day) for 12 to 24 weeks was reported to improve cognitive function [94]. Lycopene (carotene, a pigment of red and pink fruits) has anti-inflammatory, neuroprotective and antioxidant properties, in a preclinical mouse model of AD [95]. Interestingly, their levels, together with another antioxidant molecule, were found to be reduced in demented patient’s plasma levels [96]. It is, thus, accepted that the reduction of these protective factors contributes to the disease.

Other phytohormones, such as Abscisic acid (ABA), has been demonstrated to have antiglucemic effects in patients with type 2 diabetes [97]. ABA has been shown to have neuroprotective properties through its interaction with Lanthionine synthetase C-like protein 2 (LANC-2) [98] and PPAR-γ [99,100]. In addition, we have demonstrated that ABA can improve memory in an animal model of AD, reducing neuroinflammatory markers and restoring insulin-mediating molecule expression [101,102,103]. To our knowledge, ABA has not yet been tested in clinical trials.

In summary, there is a large amount of evidence indicating that nutrition and/or supplementation with nutraceuticals with anti-diabetic and anti-inflammatory properties might be a good option to avoid AD progression (see Table 3). However, longer studies with stratified human patients must be conducted to establish the therapeutic application.

## 5. Targeting the Endocannabinoid System in Preclinical Models of AD

The endocannabinoid system (ECS) is a lipid-based signaling mechanism involved in the control of neuronal and brain immune function, acting as a natural defense mechanism against pathological conditions [104]. The ECS has gained attention in AD research based on reports over the last two decades demonstrating the potential of cannabinoids to target Aβ and Tau metabolism, inflammation, mitochondrial dysfunction and excitotoxicity [105].

The ECS is composed of the G-protein-coupled cannabinoid 1 and 2 receptors (CB1 and CB2, respectively), their respective endogenous ligands *N*-arachidonil-ethanolamine (AEA) and 2-arachidonilglycerol (2-AG), the ligand synthesizing enzymes *N*-acyl-phosphatidylethanolamine (NAPE) for AEA, and 1,2-diacylglycerol lipase α (DAGLα) for 2-AG and the ligand degrading enzymes fatty acid amide hydrolase (FAAH) for AEA and monoacylglycerol lipase (MAGL) for 2-AG [106,107].

**Figure 3:**
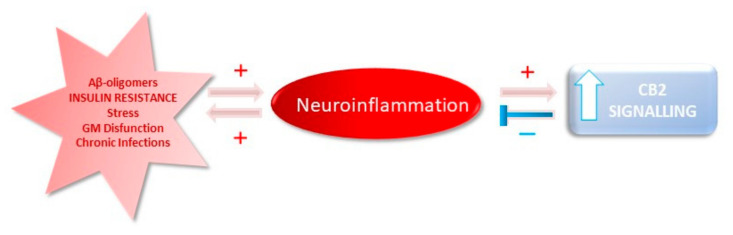
Working hypothesis, namely, the role of the ECS, on neuroinflammation in AD. Inflammatory cytokines activate CB2 signaling in microglia, which, in turn, initiates a feedback mechanism to finish this process. Due to the chronic inflammatory process in the AD brain, the CB2 pathway remain active; however, it fails to reduce inflammation.

CB1 receptors are highly expressed in the central nervous system (CNS). Data regarding the participation of the CB1 in AD are somewhat conflicting [108]. Either a reduction in the number of CB1, particularly in the frontal cortex, or no changes have been reported in AD patients [109]. In contrast, cerebral CB2 expression is sparse under normal conditions, but after specific insults (i.e., neuroinflammation), its expression augments in neurons, M1 pro-inflammatory microglia and astrocytes. CB2 cannot be detected in resting microglia, the so-called M2 phenotype [110], and the CB2 activity in M1 is postulated to facilitate M1 switch into M2 phenotype, thus initiating the anti-inflammatory and immunosuppressive responses [111]. In humans, brains from patients with AD revealed that CB2 is selectively overexpressed in cells associated to Aβ-enriched neuritic plaques and correlating with the concentrations of Aβ [112,113,114]. Activation of CB2 facilitates the removal of Aβ from frozen tissue sections of patients with AD [113]. These and other studies suggest that aiding the CB2 receptor activity could have a therapeutic potential in AD (see Figure 3).

In agreement with this hypothesis, selective synthetic CB2 agonists (JWH-15, JWH-133 and HU-308) can reduce pro-inflammatory cytokines in rodent models of AD [110,115,116]. Similar results are found with JZL184, a synthetic inhibitor of MAGL (the degrading enzyme of the CB2 ligand 2-AG) in a mouse model of AD [117].

The major natural exogenous cannabinoids are delta-9-THC (THC) and cannabidiol (CBD), mainly found in the plant *Cannabis sativa.* The THC is agonist of CB1, and has psychotropic effects, in AD patients, THC has been evaluated to treat neuropsychiatric symptoms in a randomized trial, 24 subjects were administered THC (1.5 mg/three times day) for three weeks. Although well tolerated, it had no effect on Neuropsychiatric Inventory (NPI). The authors conclude that longer treatment and well-tolerated higher doses may offer better results [67]. In contrast to THC, CBD is a non-psychoactive cannabinoid. In cellular studies, CBD reduced Aβ production and improved cell viability by inducing APP ubiquitination [118,119]. In rodent models of AD, CBD administration reduced Tau hyperphosphorylation, and significantly attenuate neuroinflammation, improving cognitive function [120,121]. Although, to our knowledge, the potential therapeutic effect of CB2 have not yet been tested in AD patients, this is now considered a promising therapeutic target in AD, given their participation in inflammatory regulation and also given the crosstalk between acetylcholine transmission and endocannabinoid function that has been revealed recently [122].

## 6. Gut Microbiota, Neuroinflammation and AD

The microbiota of the gastrointestinal tract is becoming increasingly relevant in the study of neuroinflammatory diseases, such as AD. The gut microbiota (GM) is composed of a complex ecosystem where mainly bacteria, but also fungi, archaea and viruses, reside in a symbiotic balance. The main phyla of bacteria encountered in healthy human GM are *Firmicutes* and *Bacteroidetes* (90%), and the remaining 10% contains *Actinobacteria*, *Proteobacteria*, *Fusobacteria* and *Verrucomicrobia* [123,124]. However, the composition of the microbiota varies intra-individually depending on the enterotype, body mass index, lifestyle, exercise frequency, ethnicity, age, diet and cultural habits [125].

A growing body of evidence supports that the GM maintains a close relationship with the activity of the CNS, though the microbiota-gut-brain axis. This bidirectional communication system connects the GM and CNS via the neural, immune, metabolic and endocrine pathways. The brain can influence the GM through the autonomic nervous system (noradrenaline and acetylcholine) and the hypothalamic-pituitary-adrenal axis (cortisol), controlling intestinal motility, acid and bicarbonate production, intestinal permeability and mucosal immune response. The autonomic system is in close contract with the enteric nervous system [126], which synthesizes a great number of neurotransmitters; in fact, many authors refer to GM as “The second brain” (for a review, see [127,128]). The immune and enterochromaffin cells also modulate the GM though other signaling molecules [129,130]. The alteration of these parameters can lead to changes in the composition of GM [129,130].

In turn, the metabolites and neurotransmitters (short-chain fatty acids, catecholamines, norepinephrine, 5-HT, GABA and acetylcholine and histamine, dynorphin and cytokines) synthesized in the GM can regulate CNS activity. Moreover, GM have been shown to influence the maturation, differentiation and activation of the brain immune system [131,132]. Furthermore, GM activity can also influence the blood brain barrier (BBB) permeability [133,134].

A very recent line of research postulates that the GM, by influencing the brain neuroinflammatory status, could participate in the pathogenesis of several neurodegenerative diseases, such as AD. Thus, the dysbiosis of the GM has been associated with the onset and progression of AD, in both animal models and human patients [132,135,136]. In general, in the microbiota of AD patients, the concentrations of *Firmicutes* and *Actinobacteria* are reduced, and the levels of *Bacteroidetes* are increased, compared to healthy subjects [137]. *Bacteroidetes* are gram-negative bacteria that generate lipopolysaccharides (LPS), and several species of bacteria such as *Escherichia Coli*, *Salmonella enterica*, *Salmonella typhimurium*, *Bacillus subtilis*, *Mycobacterium tuberculosis* and *Staphylococcus aureus*, which produce amyloid peptides in the gut [138]. The dysbiosis of the GM leads to an overproduction of LPS and amyloids in the gut that causes an increased permeability of both the intestinal and BBB [139]. In addition, LPS and amyloid peptides stimulate the epithelium immune cells triggering the innate immune response [140]. As a result, GM dysbiosis facilitates the penetration of pro-inflammatory molecules across the epithelium barrier into the bloodstream, from where they can reach the CNS, thereby contributing to the chronic neuroinflammation state [141,142].

Furthermore, higher levels of LPS have been found surrounding β-amyloid plaques in the brains of AD patients compared to healthy individuals [143].

It is well known that the food habits are a determining factor in the composition of the microbiota. Thus, the consumption of a varied diet rich in fiber, such as a Mediterranean diet (MD), has been shown to maintain the balance and diversity within the intestinal microbiome. A recent study in mice reveals that the administration of an MD to obese mice prevents GM dysbiosis, preserved colonic mucus barrier and reduced systemic and neuroinflammation, improving synaptic plasticity and cognitive function [144]. Moreover, in humans, adhering to an MD confers anti-inflammatory and positive regulatory properties to the microbiota and ameliorates its metabolic profile [145]. Importantly, an MD decreases the expression of some AD’s biomarkers (Tau-p181 and Aβ_42_) in the CSF of subjects with MCI [146]. Hence, maintaining a healthy microbiota by the habitual ingestion of a fiber-rich diet prevents neuroinflammation. Therefore, it is postulated that a healthy nutrition would ameliorate AD’s symptoms and possibly prevent AD progression. According to these premises, MD as an intervention strategy against AD has been tested in a six-year clinical trial with over 500 patients, aged 55–80. Although in this study no neuroinflammatory markers were found, the results in memory tasks were very promising [147].

The administration of probiotics and prebiotics has been propounded as another possible treatment to restore altered GM in AD patients. Probiotics are commonly bacteria, such as *Lactobacillus* and *Bifidobacterium*, that help fiber digestion, produce vitamins and contribute to a healthy immune system activity [148]. Several reports show that the oral ingestion of different probiotics reduces the content of pro-inflammatory cytokines in the brain and Aβ load, and improves cognitive function in murine models of AD [149,150]. In humans, a randomized double-blind placebo-controlled trial demonstrated that probiotic *Bifidobacterium breve A1* intake decreases memory disturbances in elderly people with memory complaints but not dementia [151].

Prebiotics are also food components that improve the balance of healthy bacteria. Similar to probiotics, distinct rodent models of AD treated with prebiotics (such as fructo-oligosaccharides and inulin) exhibit a GM amelioration, concomitant with a decrease in Aβ deposition, neurodegeneration and oxidative stress and an improvement in neurotransmitter secretion and cognitive abilities [152,153,154]. It is important to remark that the therapeutic effects derived from the modulation of the microbiota by the administration of pro- and prebiotics in AD from all these studies have been linked to a reduction in neuroinflammation.

Another strategy to reverse microbiota dysbiosis in AD is fecal microbiota transplant (FMT). In animal models, two studies have reported that FMT from WT to transgenic mice, recovers GM dysbiosis, while reducing neuroinflammation and enhancing synaptic plasticity together with a decrease in cognitive deficits, in an APP/PSEN mice model [155] and in an ADLP^APT^ mice model [156].

Overall, alterations in the GM are beginning to be considered as a pathophysiological mechanism in AD. GM dysbiosis heightens the permeability of the intestinal epithelial barrier and the BBB, and consequently increases the neuroinflammatory state of the organism, thereby aggravating the symptomatology of AD. In fact, some authors point out that GM dysfunction should be one of the main origins of the pathology. In this way, the restoration and maintenance of a healthy microbiota could play a key role in the prevention and treatment of AD (see Table 4). Although specific trials evaluating GM in AD have not yet been published, it is safe to assume that the consumption of a balanced diet high in fiber and low in fat and the use of pro/prebiotics will contribute to reduce neuroinflammation in AD, and therefore, aid in ameliorating its neurological and neuropsychiatric symptoms.

## 7. Conclusions

Neuroinflammation and insulin resistance are considered major neuropathological events underlying the onset and progression of AD; therefore, multiple strategies that target these processes have been developed to effectively treat this disease. In the current review, we have revised some of the latest preclinical and clinical studies targeting inflammation in AD, either directly with anti-inflammatory drugs or indirectly, improving insulin signaling.

A recent metanalysis has reviewed the results of dietary supplementation in clinical trials against AD. The results indicate that there may be an improvement in cognitive function in AD, particularly in early stages, but indicate that the effect of most dietary interventions on cognition in AD patients remains inconclusive [157]. Finally, a multidomain intervention with exercise and diet are the main strategies that showed very promising results in preventing cognitive decline in 2654 people at risk [158] (see Table 5). Again, it is not known whether this outcome would be obtained in already-diagnosed AD patients. Taking together all clinical studies revised, we conclude that strategies targeting neuroinflammation together with insulin resistance have, finally, demonstrated to be a promising therapeutic potential in AD, especially at early stages. However, many molecules have produced inconclusive results, and other methods, such as promoting neuroprotection via CB2 boosting or restoring GM, are still at the preclinical stage. In addition, patient’s stratification seems to be crucial to determine best treatment. The definite cure for AD does not exist yet; however, targeting neuroinflammation may be a path worth pursuing.

## Figures and Tables

**Table 1:** Clinical studies testing antioxidant molecules with potential therapeutic value for Alzheimer’s disease treatment. Other: other symptoms or biomarkers evaluated; NT: not tested; U: unspecified; ADAS-Cog: Alzheimer disease assessment scale–cognitive; APS: Alzheimer Progression Score; BADLS: Bristol Activities of Daily Living Scale; CGI-I: Clinical Global Impression-improvement; MMSE: Mini Mental Status Evaluation Test; NPI: Neuropsychiatry Inventory.

Compound (Dose).	Source	Patients (Years Old)	Study Design	Inflammatory/AD Biomarkers	Cognitive Effect	Other	Citation Year
Naproxen (220 mg/twice daily)	Derived from propionic acid	195 (>55)	2 years	NT	= APS progression	-	[34] 2020
Celecoxib (200 mg/twice daily)	Derived from propionic acid	2356 (70–85)	3 years	NT	= ADAPT score	-	[35] 2015
Etanercept (50 mg/once weekly subcutaneous)	U	41 (70–74)	24 weeks	= TNF-α levels = IL-6 levels = IL-10 levels = IL-12p70 levels = CRP levels	= ADAS-cog score = BADLS score = CGI-I = Cornell Scale score = MMSE score = NPI score	-	[36] 2015

**Table 2:** Clinical studies testing anti-inflammatory molecules with potential therapeutic value for Alzheimer disease treatment. Other: other symptoms or biomarkers evaluated. NT (not tested); U (unspecified). Acetate, propionate and butyrate are short-chain fatty acids. ADAS-Cog: Alzheimer disease assessment scale–cognitive; ADFACS: Alzheimer’s Disease Functional Assessment and Change Scale; ADCS-ADL: ADCS Activity of Daily Living; CDR: Clinical Dementia Rating; MMSE: Mini Mental Status Evaluation Test; NPI: Neuropsychiatric Inventory; PAL-WMS-R: Paired-Associate Learning Wechsler Memory Scale.

Compound (Dose)	Source	Patients (Years Old)	Study Design	Inflammatory/AD Biomarkers	Cognitive Effect	Other	Citation Year
Metformin (200 mg/day)	*Galega officinalis* plant	20 (55–80)	8 weeks	= Aβ42 levels	↑ Learning and memory (CANTAB PAL scale)	Crosses the BBB	[46] 2017
				= phospho-TAU levels	↑ Attention (DMS Percent Correct Simultaneous)		
				= total TAU levels			
Lactoferrin (250 mg/day)	Milk	50 (>65)	3 months	↑ IL-10 levels			[50] 2019
				↑ GSH levels		↑ Ach levels	
				↓ IL-6 levels		↑ 5-HT levels	
				↓ Aβ42 levels		↑ AKT levels	
				↓ Caspase-3 levels	↑ MMSE score	↑ phospho- AKT(S473) levels	
				↓ Cholesterol levels	↑ ADAS-Cog11 score	↑ PI3K levels	
				↓ HSP90 levels			
				↓ TAU pTAU(181)		↓ PTEN levels	
				↓ NO levels		↓ MAPK1 levels	
				↓ MDA levels			
Vitamin B12 (25 μg/day) + Folic acid (800 μg/day)	Vitamin B12: animal products Folic acid: broccoli, peas, chickpeas, leafy green vegetables	240 (>65)	6 months	↓ IL-6 levels		-	[54] 2019
				↓ TNF-α levels	↑ Full Scale IQ (FSIQ) score		
				↓ MCP-1 levels	↑ Verbal intelligence quotient (VIQ) score		
				↓ Homocysteine levels	↑ Information and Digit Span		
Vitamin B12 + Risperidone and Quetiapine (atypical antipsychotic drugs)	Risperidone: and Quetiapine are synthetic	102 (>65)	U	↑ TGF-β score ↓ IL-8 levels ↓ TNF-α levels ↓ CD68 levels	NT	↓ Pain (VAS scale)	[55,56] 2018
Nilvadipine (8 mg/day)	Pyridine (crude coal tar)	511 (>50)	18 months	NT	= ADAS-Cog 12 = CDR-sb = DAD	-	[62] 2018
Simvastatin 80 mg/day	Statins (Fungus Aspergillus terreus)	80 (>50)	18 months	↓ IL-6 levels		-	[63] 2017
				↓ IL-1β levels	↑ ADAS-Cog score		
				↓ ACT levels	↑ MMSE score		
				↓ TNF-α levels	↑ Dependence Scale score		
				↓ APP levels	↑ ADCS-ADL score		
				↓ BACE1 levels	↑ NPI score		
				↓ Aβ levels			
Simvastatin 40 mg/day		406 (>50)	18 months	↓ CRP levels	= ADAS-Cog score	↑ HDL levels	[64] 2011
					= MMSE score		
					= Dependence Scale score	↓ Total cholesterol levels	
					= ADCS-ADL score		
					= NPI score	↓ LDL levels	
Atorvastatin 40 mg/day	Statins	178 (45–60)	18 months	↓ IL-1β levels ↓ IL-6 levels ↓ TNF-α levels ↓ CRP levels ↓ MCP-1 levels	NT	↓ Lipid levels	[65] 2016
Atorvastatin 80 mg/day		640 (50–90)	18 months	NT	= ADAS-Cog score		[66] 2010
					= ADCS-CGIC score	↓ Total cholesterol levels	
					= MMSE score		
					= CDR-SB score	↓ LDL-C levels	
					= ADFACS score	↓ Triglycerides levels	
					= NPI score		
Tetrahydrocannabinol (4.5 mg/day)	*Cannabis* plant	50 (78–79)	3 weeks	NT	= NPI score = Cohen-Mansfield Agitation Inventory = Quality of Life-Alzheimer’s Disease = Barthel Index score = PAL WMS-R score		[67] 2015

**Table 3:** Clinical studies testing nutraceuticals with a potential therapeutic value for Alzheimer’s disease treatment. Other: other symptoms or biomarkers evaluated; NT: not tested; U: unspecified; ADAS-Cog: Alzheimer disease assessment scale–cognitive; ADCS-ADL: ADCS Activity of Daily Living; AVL: Auditory Verbal Learning Test; CCR: Cambridge Contextual Reading Test; CN: Category naming test; COWA: Controlled Oral Word Association Test; DSS: Digit Symbol Substitution Test; MMSE: Mini Mental Status Evaluation Test; MoCA: Montreal Cognitive Assessment; WAIS-R: Wechsler Adults Intelligence Scale.

Compound (Dose)		Source/Study	Patients (Years Old)	Study Design	Inflammatory/AD Biomarkers	Cognitive Effect	Other	Citation
Curcumin	(1.5–4 g/day)	Turmeric	34 (>50)	6 months	↓ Aβ aggregation	= MMSE score	-	[72] 2008
	(1500 mg/day)		160 (40–90)	12 months	NT	↑ MoCA score = CCR Test score = DASS score = AVL Test score = COWA Test score = WAIS-R score = CogState score	-	[73] 2016
Resveratrol (500 mg/day)		Red grapes, peanuts and other plant species	119 (>49)	52 weeks	↑ MDC levels ↑ IL-4 levels ↑ FGF-2 levels ↑ MMP10 levels ↓ MMP9 levels ↓ IL-12 levels ↓ RANTES levels	↑ ADCS-ADL score	-	[76,77] 2015
PUFA 800 mg docosahexaenoic acid 225 mg eicosapentaenoic acid/day.		Multimodal The MAPT study	1680 Non demented (>70)	3 years	NT	= MMSE score = DSS Test score = CN Test	Safe	[91] 2017
Heat processed Ginseng (4.5 g/day)			40 (U)	6 months	NT	↑ ADAS-Cog score ↑ MMSE score	-	[94] 2012
Abscisic acid (40/80 µg)		Fig fruit extract	10 Non-demented (18–45)	4 non-consecutive sessions	NT	NT	Safe ↓Postprandial glycemic responses.	[97] 2019

**Table 4:** Clinical studies focused on the regulation of the activity of the gut microbiota as a potential treatment against Alzheimer’s disease. Other: other symptoms or biomarkers evaluated; NT: not tested; CDT: clock Drawing test; MMSE: Mini-Mental Status; RBANS: Repeatable Battery for the Assessment of Neuropsychological Status.

Compound (Dose)	Patients (Years Old)	Study Design	Inflammatory/AD Biomarkers	Cognitive Effect	Other	Citation Year
Mediterranean-Ketogenic diet (MMKD) (<10% carbohydrate, 60–65% fat, and 30–35% protein) American Heart Association diet (AHAD) (55–65% carbohydrate, 15–20% fat, and 20–30% protein)	17 (64.6 ± 6.4)	6 weeks	↓ Aß42 ↓ Tau-p181	NT	↑ *Enterobacteriaceae, Akkermansia, Slackia, Christensenellaceae* and *Erysipelotriaceae* ↑ Propionate and butyrate ↓ *Bifidobacterium* and *Lachnobacterium* ↓ Fecal acetate and lactate	[146] 2019
			= Aß-42 = Tau-p181	NT	↑ *Mollicutes* ↑ Acetate and propionate ↓ Butyrate	
Mediterranean diet (extra-virgin olive oil 1 l/week) or 30 g/day nuts	522 (55–80)	6.5 years	NT	↑ MMSE score ↑ CDT score	-	[147] 2013
*Bifidobacterium breve* A1 (daily)	117 (50–80)	12 weeks	NT	↑ RBANS score ↑ MMSE score	Safe	[151] 2019

**Table 5:** Clinical studies testing a multidomain intervention as a treatment against Alzheimer’s disease. Other: other symptoms or biomarkers evaluated; NT: not tested; NTB; Neuropsychological Test Battery.

Compound (Dose) and Treatment	Source	Patients (Years Old)	Study Design	Inflammatory/AD Biomarkers	Cognitive Effect	Other	Citation
Nutritional intervention (10–20% of daily energy (E%) from proteins, 25–35E% from fat, 45–55 E% from carbohydrates, 25–35 g/day dietary fiber) + Physical exercise training + Cognitive training (executive processes, working memory, episodic memory and mental speed) + Social activities	Multimodal The FINGER study	1260 (60–77)	2 years	NT	↑ NTB score ↑ NTB Executive functioning domain score ↑ NTB Processing speed domain score = NTB Memory domain score	-	[158] 2015

## Abbreviations

2-AG	2-arachidonilglycerol
5-HT	5-hydroxytryptamine (serotonin)
Aβ	Amyloid beta
ABA	Abscisic acid
AD	Alzheimer’s disease
ADAS-Cog	Alzheimer disease assessment scale–cognitive
ADAPT	Alzheimer’s disease anti-inflammatory prevention trial
ADCS-ADL	Alzheimer’s disease cooperative study-activities of daily living
AEA	*N*-arachidonil-ethanolamine
AMPK	Adenosine monophosphate-activated protein kinase
APP	Amyloid protein precursor
APS	Alzheimer progression score
AVL	Auditory Verbal Learning Test
BACE1	β-site APP-cleaving enzyme 1 secretase
BADLS	Bristol Activities of Daily Living Scale;
BBB	Blood brain barrier
BDNF	Brain-derived neurotrophic factor
CB1	G-protein-coupled cannabinoid 1 receptor
CB2	G-protein-coupled cannabinoid 2 receptor
CBD	Cannabidiol
CCR	Cambridge Contextual Reading Test
CD68	Cluster of Differentiation 68
CDT	Clock Drawing Test
CGI-I	Clinical Global Impression-improvement
CN	Category naming test
CNS	Central nervous system
COX	Cyclooxygenase
COWA	Controlled Oral Word Association Test
CSF	Cerebrospinal fluid
DAGLα	Diacylglycerol Lipase alpha
DAMPs	Damage-associated molecular patterns
DMS	Delayed Matching to Sample
DSS	Digit Symbol Substitution
ECS	Endocannabinoid system
EGCG	Epigallocatechin gallate
FAAH	Fatty acid amide hydrolase
FMT	Fecal microbiota transplant
GABA	Gamma-aminobutyric acid
GM	Gut microbiota
IFN-γ	Interferon gamma
IL-1β	Interleukin 1 beta
IN	Intranasal
IRS1	Insulin receptor substate 1
LanCL2	Lanthionine synthetase *C*-like protein 2
LDL	Low density lipoprotein
LPS	Lipopolysaccharides
MAGL	Monoacylglycerol lipase
MAPT	MultiDomain Alzheimer preventive trial
MCI	Mild cognitive impairment
MD	Mediterranean diet
MMSE	Mini-mental state examination
MoCA	Montreal Cognitive Assessment
mTOR	Mammalian target of rapamycin
NAPE	*N*-acyl-phosphatidylethanolamine
NTB	Neuropsychological Test Battery
NTB	Neuropsychological Test Battery NFTs Neurofibrillary tangles
NGF	Nerve growth factor
NLR	NOD-like receptors
NLRP3	NLR family pyrin domain containing 3
NPI	Neuropsychiatric Inventory
NSAIDs	Non-steroidal anti-inflammatory drugs
p70S6K	p70 ribosomal S6 kinase
PAL	Paired associate learning scale
PAMPs	Pathogen-associated molecular patterns
PEA	Palmitoylethanolamide
PG	Prostaglandin
PPAR-α	Peroxisome proliferator-activated receptor alpha
PRRs	Pattern recognition receptors
PUFAs	Polyunsaturated fatty acids
RANTES	Regulated on Activation, Normal T cell Expressed and Secreted
RBANS	Repeatable Battery for the Assessment of Neuropsychological Status
RV	Resveratrol
SMMSE	Standardized mini-mental state examination
T2DM	Type 2 diabetes mellitus
TGF-β	Transforming growth factor beta
THC	Tetrahidrocannabinol
TLRs	Toll-like receptors
TNF-α	Tumor necrosis factor alpha
WAIS-R	Wechsler Adults Intelligence Scale
WMS-R	Wechsler Memory Scale
WT	Wild-type
